# Prevalence of Human Papillomavirus‐Associated Head and Neck Cancer in Rwanda: A 10‐Year Review

**DOI:** 10.1002/cam4.70423

**Published:** 2024-11-12

**Authors:** Fidel Rubagumya, Lydia Businge, Wilma H. Hopman, Gad Murenzi, Aline Uwimbabazi, Vincent Kwizera, Julienne Imuragire, Thierry Z. Muvunyi, Isabelle Izimukwiye, Adebola Adedimeji, Rachael E. Barney, Gregory J. Tsongalis, Mary D. Chamberlin, Kathryn Anastos, Rafi Kabarriti

**Affiliations:** ^1^ Rwanda Military Hospital Kigali Rwanda; ^2^ Research for Development (RD Rwanda) Kigali Rwanda; ^3^ Dartmouth Cancer Center Lebanon New Hampshire USA; ^4^ Department of Oncology University of Rwanda Kigali Rwanda; ^5^ Cancer Unit, Rwanda Biomedical Center Kigali Rwanda; ^6^ Department of Public Health Sciences Queen's University Kingston Ontario Canada; ^7^ King Faisal Hospital Kigali Rwanda; ^8^ Department of Epidemiology & Population Health Albert Einstein College of Medicine Bronx New York USA; ^9^ Department of Medicine and Epidemiology & Population Health Montefiore Einstein Cancer Center Bronx New York USA; ^10^ Department of Radiation Oncology Montefiore Einstein Cancer Center Bronx New York USA

**Keywords:** Africa, head and neck cancers, HPV, LMIC, Rwanda

## Abstract

**Introduction:**

Head and neck cancer (HNC) is a significant global health burden, with late presentation leading to complex treatment. While human papillomavirus (HPV) infection has been implicated in HNC, data from low‐ and middle‐income countries (LMICs) are limited. In this study, we investigated the prevalence and role of HPV in head and neck cancers diagnosed in Rwanda.

**Methods:**

A retrospective cross‐sectional study was conducted using Rwanda Cancer Registry from January 2011 through December 2020. p16 immunohistochemistry as a surrogate for HPV was performed on a randomly selected case. p16‐positive cases were genotyped.

**Results:**

A total of 1001 patients with HNC were identified; 82% (*n* = 819) had squamous cell carcinoma. The mean age at diagnosis was 51.1 years, with a majority being males (58%). Oral cavity and lip (27%) were the most common primary cancer sites. Stage was unknown in most cases (75%, *n* = 747). HIV status was known in 33% (*n* = 334) of patients with 10% (*n* = 33) HIV‐positive; 22% of 202 randomly selected cases were p16‐positive; 34% of the p16‐positive cases were oropharynx. PCR analysis of p16‐positive cases showed 19% HPV positivity, and HPV16 was the most common high‐risk HPV strain, and 55.5% were recorded HPV‐positive by PCR.

**Conclusions:**

HNC cases in Rwanda have been increasing from 2011 to 2020, with a significant portion being HPV‐positive. Strategies to implement routine testing for p16, especially in oropharynx cancer patients, improved preservation of tissue samples, collection of comprehensive information including cancer risk factors, staging, and treatment are needed in Rwanda.

## Graphical abstract

High prevalence of squamous cell carcinoma in head and neck cancer (HNC) cases in Rwanda, with the oral cavity and lip being predominant sites. A significant proportion of HNC cases was linked to human papillomavirus infection, particularly in oropharyngeal cancers. Limited staging information underscores the need for improved data collection methods and comprehensive patient information.

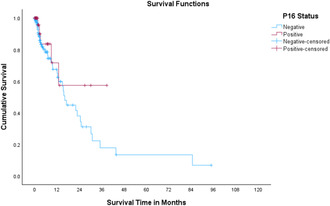

**Abbreviations**
BCCOE: Butaro Cancer Center of Excellence
BUTH: Butare University Teaching Hospital
CAP: College of American Pathologists
DNA: deoxyribonucleic acid
FFPE: formalin‐fixed paraffin‐embedded
HIV: human immunodeficiency virus
HNC: head and neck cancer
HNSCC: head and neck squamous cell carcinomas
HPV: human papillomavirus
HRP: horse radish peroxidase
KFH: King Faisal Hospital
KUTH: Kigali University Teaching Hospital
LMIC: low‐ and middle‐income countries
PCR: polymerase chain reaction
RMH: Rwanda Military Hospital
RNCR: Rwanda National Cancer Registry
RNEC: Rwanda National Ethics Committee
SSA: sub‐Saharan Africa
TNM: tumor, node, metastasis

## 1 Introduction

Worldwide, head and neck cancer (HNC) constitutes one of the most common cancers, with a global incidence of 660,000 new cases and 325,000 fatalities per year [1]. Head and neck squamous cell carcinomas (HNSCCs) are the most commonly found histological diagnoses in the oral cavity, oropharynx, hypopharynx, and larynx [2]. Because of their proximity to important head and neck structures, head and neck malignancies pose difficulty due to the anatomical nature of their sites of occurrence. The majority of these HNCs present late, making early detection or screening for these cancers challenging. Common presentations include breathing difficulties, obstructive swellings, and difficult deglutition [3]. As they are discovered at locally advanced stages, most HNCs necessitate complicated treatment regimens involving radiation therapy, chemotherapy, and/or surgery [4].

Although HNSCCs have long been linked to exposure to alcohol and tobacco, new evidence over recent years has shown that human papillomavirus (HPV) infection is responsible for a rising number of head and neck cancers [5], with HPV currently being the main contributor to oropharyngeal cancers in Northern Europe and America [6]. In Rwanda, the prevalence of uterine cervix HPV is 34%; it peaks in women under the age of 19 (54%) and declines to 20% in those over the age of 50, with HPV16 being the most prevalent type [7]. However, little is known about the HPV status in the head and neck region and more so in head and neck cancers. Rwanda has been successful in implementing a national HPV vaccination program for girls; however, boys are not vaccinated [8].

A recent study by Okerosi et al. [9], which systematically reviewed HPV‐associated HNCs in sub‐Saharan Africa (SSA) in 31 studies and 3850 patients, reported that the percentage of patients who tested positive for p16 overall was 13.6% (41 of 1037 patients), with oropharyngeal malignancies having the highest percentage (20.3%, 78 of 384 individuals). Furthermore, among the 369 HPV strains detected, the most common genotypes were HPV 16, HPV 18, and HPV 56. Of these, HPV 16 had the highest prevalence at 59.2% [9].

Although significant efforts have been made to characterize HNCs associated with HPV in developed nations, there has been little validation of these findings in low‐ and middle‐income countries (LMICs). The purpose of this study was to investigate the incidence of head and neck cancers over a 10‐year period and perform HPV testing on a small cohort to examine its prevalence in head and neck cancers in Rwanda.

## 2 Methods and Materials

This was a cross‐sectional study of patients diagnosed with head and neck cancer in Rwanda identified retrospectively from the Rwanda National Cancer Registry (RNCR) over 10 years. Rwanda is a country with 26,798 km^2^, with four major referral hospitals and one level two referral cancer center with the capacity for cancer care from diagnosis to follow‐up. Three of these hospitals, including Kigali University Teaching Hospital (KUTH), Rwanda Military Hospital (RMH), and King Faisal Hospital (KFH), are in the capital city, while one, the Butare University Teaching Hospital (BUTH), is in the south, and another, the Butaro Cancer Center of Excellence (BCCOE), is in the north of the country.

Using the RNCR, all cases assigned a diagnosis of any head and neck cancer subsite with histological confirmation were retrieved. Patients with non‐head and neck malignancies, nonsquamous cell tumors (such as lymphomas), head and neck metastases, and patients without any records or pathologic specimens at the four referral hospitals were not included in this study. For all included cases, the following variables were tabulated: Age, gender, date at diagnosis, disease subsite of head and neck, tumor, node, metastasis stage, histology, tumor grade, as well as date and status at last contact. Information that was missing from the cancer registry was retrieved from patient files at the mentioned hospitals.

We performed immunohistochemistry testing using p16 as a surrogate for HPV on 202 randomly selected cases with squamous cell carcinoma. The number was based on fund availability. Formalin‐fixed paraffin‐embedded (FFPE) tissue blocks were serially cut into thin sections of 5 μm and were prepared on a clean glass slide. Sections from paraffin blocks were then stained for p16 and scored using standards established by the College of American Pathologists (CAP). A subset of positive cases was then assessed using the polymerase chain reaction (PCR). The study was approved by the Rwanda National Ethics Committee (RNEC) and the respective institutional review boards, and as this was a retrospective study, informed consent was waived.

### 2.1 p16 Antibody Staining and Interpretation

Immunohistochemical analysis was performed on both patient tissue samples and positive control tissues using p16 INK4(JC2) mouse monoclonal antibody (Merck KGaA, Darmstadt, Germany). Tissue sections underwent heat‐induced epitope retrieval and were subsequently treated with anti‐P16 antibody and HRP (horse radish peroxidase). DAB‐chromogen was applied to visualize the target antigen recognized by the primary antibody, resulting in a brown coloration. Counterstaining was performed using Gill's hematoxylin, and slides were prepared for reading and conservation through dehydration, clearing, and mounting in a permanent mounting medium. Two senior pathologists reviewed the p16‐stained slides independently and discussed any discrepancies.

### 2.2 HPV Genotyping

Formalin‐fixed, paraffin‐embedded (FFPE) tissues from cases that tested positive for p16 were sent to the Dartmouth Cancer Center (Lebanon, New Hampshire, USA) for further testing using PCR to determine the high‐risk HPV type. DNA was extracted from FFPE tissue sections using the QIAamp DNA FFPE Tissue kit on the QIAcube instrument and quantified using Qubit dsDNA High‐Sensitivity Assay (Invitro‐gen, Waltham, MA, USA). High‐risk HPV genotyping was performed using the MeltPro High‐Risk HPV Genotyping Assay (QuanDx, San Jose, CA, USA) and the ZSLAN real‐time PCR instrument as previously described [10, 11]. Genotype results were based on melt curve analysis for each of the 14 high‐risk HPV types, and each run included positive and negative controls provided with the kit to ensure successful amplification and melt curves.

### 2.3 Data Analysis

**FIGURE 1:**
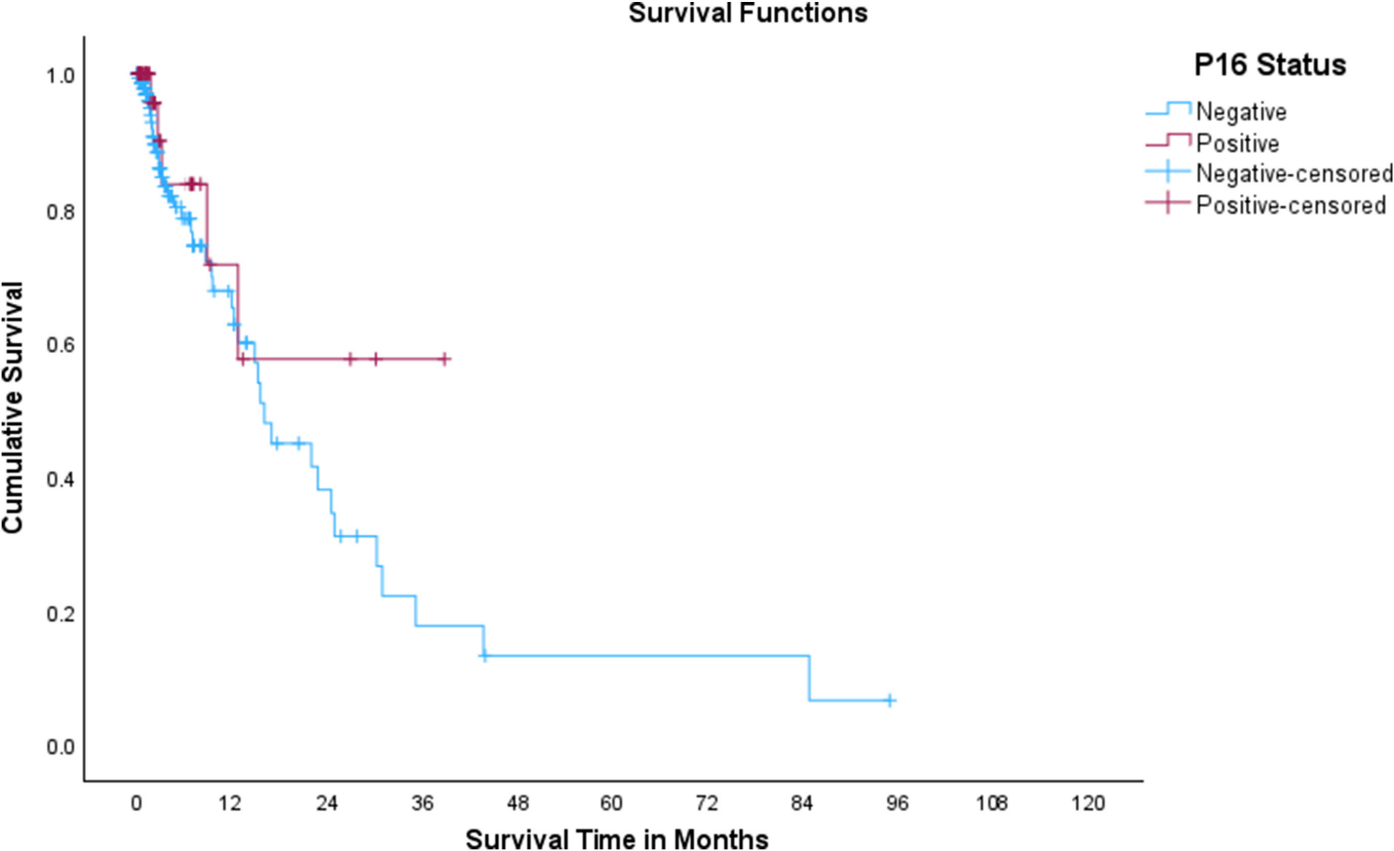
Kaplan–Meier curve for those who were p16‐negative and p16‐positive.

Data were collected in an Excel spreadsheet and imported into IBM SPSS (version 28.0 for Windows, Armonk, New York, 2022) for analysis. Data were initially analyzed using descriptive statistics, including frequencies and percentages for categorical data and means (standard deviations) for continuous data. Pearson's Chi‐square tests or the Fisher's exact test (depending on the cell size) were utilized to compare the categorical data for those who were p16‐positive and p16‐negative, while the independent samples' *t* test was used to compare continuous data. A Kaplan–Meier curve with the Log‐Rank test was used to compare survival in those who were p16‐positive and p16‐negative (Figure 1). A *p*‐value of < 0.05 was statistically significant, and no adjustment was made for multiple comparisons.

## 3 Results

### 3.1 Patients' Characteristics

**FIGURE 2:**
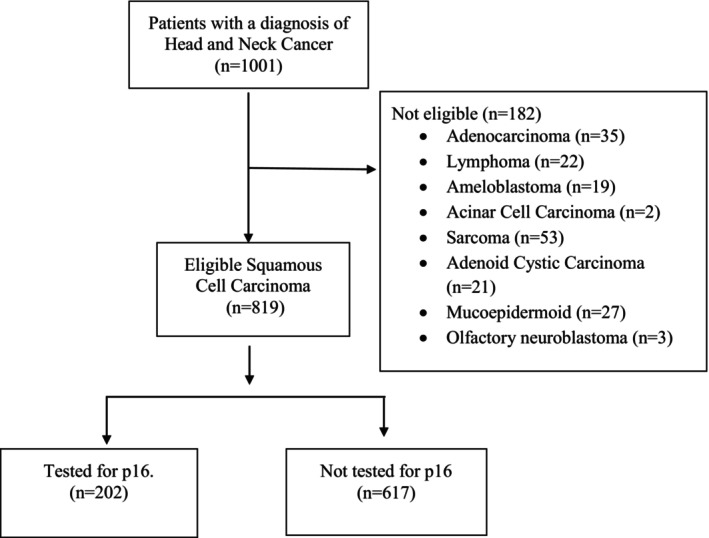
Results of head and neck cancer diagnosis by histological subtypes (*n* = 1001).

A total of 1001 patients with a histology of any head and neck subsite were identified, with 82% (*n* = 819) of patients having squamous cell carcinoma histology. Overall, the mean age at diagnosis was 51.1 (SD ± 20.0) (Table 1). Most of the patients were male (58%, *n* = 576) (Table 1). Most patients resided in the Eastern Province (26%, *n* = 265), while some came from outside Rwanda (Table 1). HIV status was known in 33% (*n* = 334) of patients, with 90% (*n* = 301) being HIV‐negative while 10% (*n* = 33) HIV‐positive, and 667 had an unknown HIV status (Table 1). The number of diagnosed cases in the cancer registry increased every year from 44 cases in 2011 to 159 cases in 2020 (Figure 2).

**TABLE 1:** Characteristics of patients diagnosed with head and neck cancers in Rwanda (*n* = 1001).

Patient characteristics	All patients (*n* = 1001)	p16‐positive (*n* = 44)	p16‐negative (*n* = 158)	*p*
Age (year), mean ± SD	51.09 ± 19.98	56.55 ± 13.52	62.23 ± 13.01	0.01
Gender, *n* (%)				0.11
Male	576 (57.5)	37 (84.1)	114 (72.2)	
Female	425 (42.5)	7 (15.9)	44 (27.9)	
Place of residence				
East	265 (26.5)	14 (32.6)	52 (33.1)	
Kigali	241 (24.1)	13 (30.2)	25 (15.9)	
South	145 (14.5%)	6 (13.9)	24 (15.3)	
West	142 (14.2%)	4 (9.3)	25 (15.9)	0.05
North	123 (12.3%)	3 (6.9)	26 (16.6)	
Unknown	77 (7.7%)	2 (4.7)	1 (0.6)	
Burundi	4 (0.4%)	0 (0)	4 (2.6)	
Congo	2 (0.2%)	1 (2.3)	0 (0)	
Cancer site, *n* (%)				
Oral cavity and lip	268 (26.8%)	14 (31.8)	81 (51.3)	
Larynx	242 (24.2%)	1 (2.3)	33 (20.9)	< 0.001
Nasal and paranasal sinuses	105 (10.5%)	2 (4.6)	8 (5.1)	
Salivary glands	102 (10.2%)	5 (11.4)	5 (3.2)	
Nasopharynx	91 (9.1%)	1 (2.3)	5 (3.2)	
Mandible	89 (8.9%)	5 (11.4)	7 (4.4)	
Oropharynx	76 (7.6%)	15 (34.1)	14 (8.9)	
Hypopharynx	28 (2.8%)	1 (2.3)	5 (3.2)	
Stage, *n* (%)				
I	35 (3.5%)	3 (6.8)	7 (4.4)	
II	61 (6.1%)	0 (0)	15 (9.5)	
III	73 (7.3%)	6 (13.6)	15 (9.5)	0.02
IV	85 (8.49%)	0 (0)	15 (9.5)	
Unknown	747 (74.6)	35 (79.5)	106 (67.1)	
Grade, *n* (%)				
I	193 (19.3)	15 (34.1)	70 (44.3)	
II	137 (13.7)	10 (22.7)	55 (34.8)	0.04
III	65 (6.5)	5 (11.4)	8 (5.1)	
IV	58 (5.7)	1 (2.3)	1 (0.6)	
Unknown	548 (54.8)	13 (29.6)	24 (15.2)	
HIV, *n* (%)				
Yes	33 (3.3)	2 (4.6)	5 (3.2)	0.41
No	301 (30.1)	11 (25)	56 (35.4)	
Unknown	667 (66.6)			

### 3.2 Disease Characteristics

The most common primary cancer sites were oral cavity and lip (27%, *n* = 268), followed by larynx (24%, *n* = 242), nasal and paranasal sinuses (10%, *n* = 105), salivary gland (10%, *n* = 102), and nasopharynx (9% *n* = 91), while oropharynx had 8% (*n* = 79) of the cases (Table 1). Stage was unknown in most cases (75%, *n* = 747); for those with known stage, stage IV was the most common (8%, *n* = 85/747), followed by stage III (7%, *n* = 73/747), stage II (6%, *n* = 61/747), and stage I (4%, *n* = 35/747). The most common histology was squamous cell carcinoma with 82% (*n* = 819) of cases. Grade was known in a total 45% (*n* = 453) cases, with 43% (*n* = 193/453) of them being grade I, 30% (*n* = 137/453) grade II, 14% (*n* = 65/453) grade III, and 13% (*n* = 58/453) as grade IV (Table 1).

### 3.3 P16 Status

Due to financial constraints, p16 is not routinely tested in clinical care; we tested 20% (*N* = 202/1001) for p16 as a surrogate for HPV. Among these, 22% (*n* = 44/202) were positive and 78% (*n* = 158/202) were negative. Of the positive P16, 84% (*n* = 37/44) were male (Table 1).

The p16 status of the patients varied by the disease site. Among the 44 positive cases, 34% (15/44) were oropharyngeal, and 32% were cancers of oral cavity and lip (14/44) (Table 1). Age, province, disease primary site, stage, and grade were all statistically significant predictors of p16 positivity, but gender and HIV status were not (Table 1).

Of the 202 p16‐tested cases, the highest percent of p16 positivity was 51.7% among patients with oropharyngeal cancer, followed by 50% for salivary gland, 41.7% for mandible, 20% for nasal and paranasal sinuses, 16.7% for nasopharynx and hypopharynx, 14.7% for oral cavity and lip, and 2.9% for larynx cancers.

### 3.4 High‐Risk HPV Status by Genotyping

**FIGURE 3:**
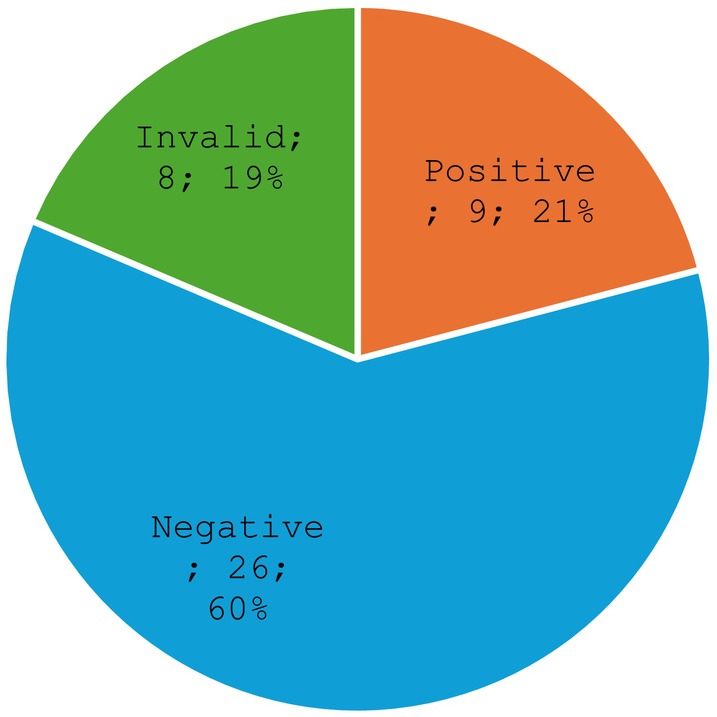
HPV genotyping of head and neck cancers.

Figure 3 and Table 2 show results of HPV genotyping. Of the 43 samples tested, 21% (*n* = 9/43) tested positive for HPV, 61% (*n* = 26/43) tested negative, and 19% (*n* = 8/43) cases were invalid. Among those that tested positive for HPV, 67% were genotype HPV 16 (14% of total HPV‐tested samples), 11% were HPV 31 (2.3% of the total HPV‐tested samples), 11% were HPV 33 (2.3% of total HPV‐tested samples), and 11% were HPV 45 (2.3% of total HPV‐tested samples). Among the nine PCR‐HPV‐positive cases, 55.5% (*n* = 5/9) were oropharynx, and all were male with the average age of 57 years, while 2 were mandible, 1 nasal cavity, and 1 parotid (Table 3).

**TABLE 2:** HPV subtypes.

Genotype	Count	% of genotype positive	% of total samples
HPV 16	6	66.7	14.0
HPV 31	1	11.1	2.3
HPV 33	1	11.1	2.3
HPV 45	1	11.1	2.3
HPV 18	0	0	0
HPV 35	0	0	0
HPV 39	0	0	0
HPV 51	0	0	0
HPV 52	0	0	0
HPV 56	0	0	0
HPV 58	0	0	0
HPV 59	0	0	0
HPV 66	0	0	0
HPV 68	0	0	0

**TABLE 3:** HPV‐positive disease subsites.

Disease site	Numbers	Percentage
Oropharynx	5	55.5
Nasal cavity	1	0.1
Mandible	2	22.2
Parotid gland	2	22.2

### 3.5 Overall Survival

**FIGURE 4:**
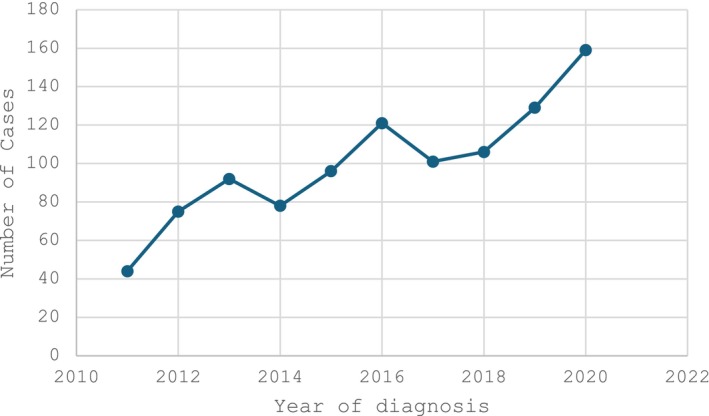
Head and neck diagnoses over a period of 12 years (*n* = 1001).

At the time of the last follow‐up, a total of 79% (*n* = 793) were still alive. The overall median survival time across the studied cancer sites was 24 months, with a 95% confidence interval ranging from 18 to 30 months, which is low since the vast majority was censored within the first 3 years. The median survival time among the p16‐tested group was undefined for positive p16 patients since an insufficient number reached the threshold for this to be calculated, while it was 16 months for p16‐negative patients, with the vast majority censored within the first 18 months (Figure 4). The Log Rank (Mantel‐Cox) test showed no statistically significant difference in survival time between negative and positive p16 patients (*p* = 0.237).

## 4 Discussion

In this first study describing patients with head and neck cancers in Rwanda, we have explored the epidemiology of these cancers, with several findings. First, the mean age at diagnosis of head and neck cancers in Rwanda was 51 years. Second, there is a higher incidence of head and neck cancers in males. Third, the distribution of head and neck cancer cases varied across different subsites. The most common primary sites were the oral cavity and lip, followed by the larynx, nasal and paranasal sinuses, and salivary glands. Fourth, the number of diagnosed HNC cases has been increasing over the last 10 years. Fifth, HPV plays a significant role in HNC, accounting for 22% of cases with squamous cell carcinoma histology and more than half of patients having HPV‐associated oropharynx cancer. Lastly HPV 16 is the most common high‐risk HPV type in HNCs in Rwanda.

Our findings should be interpreted in the context of existing literature on head and neck cancers, in alignment with previous studies in different regions suggesting that head and neck cancers predominantly affect individuals in their fifth and sixth decades of life [12, 13]. Additionally the apparent gender disparity is consistent with global trends and can be attributed to a higher prevalence of risk factors such as tobacco and alcohol use among males [14, 15]. Subsite disease patterns in this study are in line with previous studies reporting similar site‐specific distributions in Africa where the most common subsite is oral cavity and lip with 27% and laryngeal cancer with 24% of HNC cases [9, 13, 16]. However, it is worth noting that these findings differ from Western populations, where oropharyngeal cancers, often associated with HPV infection, are more prevalent [17, 18]. Stage distribution in our study is similar to another study in which the highest proportion was stage III, followed by stage IV [19]. In terms of HPV‐associated HNCs, studies have reported the proportion of HPV‐related HNCs to be 3% [13], 31.5%, and 12.2% [20, 21], and our study findings fall among these percentages. The limited availability of p16 testing as a surrogate for HPV highlights a challenge faced in many low‐resource settings. Only 20% of the patients in our study were tested for p16 due to financial constraints. This limitation hinders our ability to accurately assess the role of HPV in head and neck cancers in Rwanda and to provide more accurate staging using HPV information. However, the proportions of p16 positivity observed in our study, particularly in the oropharynx and oral cavity, are consistent with studies from the SSA region, which were found to be 20.3% [9] and 20% [22]. In our study, we observed cases of p16 positivity in salivary glands with 11%. However, this is not uncommon as a study by Brunner et al. found that among 38 cases of minor salivary gland, 71% of samples expressed p16. However, none showed low‐risk HPV DNA, but high‐risk HPV DNA was found in two samples [23]. Additionally, our findings are in line with other studies in terms of the most common high‐risk HPV type being HPV 16 [9].

Despite the valuable insights gained from this study, several limitations should be acknowledged. First, the retrospective nature of the study and the reliance on data from the cancer registry introduce potential biases such as misclassification and missing information. Second, the lack of comprehensive staging and grading data limited our ability to assess disease severity accurately. Third, discrepancy between p16‐positive and HPV genotyping results can be attributed to DNA degradation secondary to how samples/FFPE were preserved or stored or could be a biological issue with the virus and highlights the need for improved infrastructure and sample‐handling protocols in future studies. Additionally, there could be p16 upregulation which can justify the discrepancies between p16 results versus those of HPV genotyping by PCR. However, these other mechanisms could not be ascertained. Fourth, the follow‐up for most patients was short, limiting our ability to assess survival. Fifth, our study lacks data on risk factors such as tobacco and alcohol and treatment modalities used. Despite these limitations, our study contributes to the limited body of literature on head and neck cancers in Africa, particularly in the Rwandan context.

Based on our findings, several policy implications can be drawn for Rwanda and SSA. First, there is a need to improve access to diagnostic tools, including p16 testing and HPV detection methods, in low‐resource settings like Rwanda. Strengthening laboratory infrastructure and ensuring affordability of these tests can enhance early detection and appropriate management of HPV‐related head and neck cancers. Collaboration with international partners and organizations can support capacity‐building efforts and technology transfer. Second, given the site‐specific distribution of head and neck cancers observed in our study, targeted prevention strategies should be implemented. This may include promoting oral hygiene practices, encouraging regular dental check‐ups, and raising awareness about the signs and symptoms of these cancers. Additionally, efforts should be made to increase HPV vaccination coverage in both males [24] and females, as it has shown promise in reducing the incidence of HPV‐associated head and neck cancers in other populations [25, 26]. Third, while we did not collect data on risk factors with the available evidence of the role of tobacco and alcohol as risk factors for head and neck cancers, efforts should be directed toward raising awareness about the risk factors associated with head and neck cancers, with a particular emphasis on addressing tobacco and alcohol use among males, as they represent a high‐risk group. Public health campaigns should focus on promoting smoking cessation programs and alcohol moderation to reduce the incidence of these cancers. Finally, improving cancer surveillance systems, such as cancer registries, is crucial for monitoring trends, evaluating interventions, and guiding resource allocation. Adequate funding and training should be provided to ensure accurate and comprehensive data collection, including detailed demographics, risk factors, staging and treatment modalities information, to facilitate a better understanding of the burden and outcomes of head and neck cancers in Rwanda.

## 5 Conclusions

In conclusion, this study provides valuable insights into the epidemiology and characteristics of head and neck cancers in Rwanda. The findings underscore the importance of targeted prevention strategies, addressing gender disparities, improving access to diagnostic tools, and strengthening cancer surveillance systems. By implementing evidence‐based policies and interventions, Rwanda, and other countries in SSA can make significant strides in reducing the burden of head and neck cancers and improving patient outcomes. Continued research and collaboration are essential to further our understanding of these cancers in the African context and develop effective strategies to combat them.

## Author Contributions

**Fidel Rubagumya:** conceptualization (lead), data curation (lead), validation (lead), writing – original draft (lead), writing – review and editing (lead). **Lydia Businge:** data curation (equal), validation (equal), writing – review and editing (equal). **Wilma H. Hopman:** formal analysis (lead), validation (equal). **Gad Murenzi:** validation (equal), writing – review and editing (equal). **Aline Uwimbabazi:** data curation (equal), writing – review and editing (equal). **Vincent Kwizera:** data curation (equal), validation (equal), writing – review and editing (equal). **Julienne Imuragire:** data curation (equal), validation (equal), writing – review and editing (equal). **Thierry Z. Muvunyi:** data curation (equal), validation (equal), writing – review and editing (equal). **Isabelle Izimukwiye:** data curation (equal), validation (equal), writing – review and editing (equal). **Adebola Adedimeji:** validation (equal), writing – review and editing (equal). **Rachael E. Barney:** data curation (equal), validation (equal), writing – review and editing (equal). **Gregory J. Tsongalis:** data curation (equal), validation (equal), writing – review and editing (equal). **Mary D. Chamberlin:** validation (equal), writing – review and editing (equal). **Kathryn Anastos:** validation (equal), writing – review and editing (equal). **Rafi Kabarriti:** conceptualization (supporting), writing – original draft (supporting), writing – review and editing (equal).

## Conflicts of Interest

The authors declare no conflicts of interest.

## Data Availability

The data that support the findings of this study are available on request from the corresponding author.

## References

[1] F. Bray , J. Ferlay , I. Soerjomataram , R. L. Siegel , L. A. Torre , and A. Jemal , “Global Cancer Statistics 2018: GLOBOCAN Estimates of Incidence and Mortality Worldwide for 36 Cancers in 185 Countries,” CA: A Cancer Journal for Clinicians 68, no. 6 (2018): 394–424, 10.3322/caac.21492. 30207593

[2] J. Pfeiffer , T. Wiech , W. Maier , G. J. Ridder , R. Laszig , and R. Birkenhäger , “Head and Neck Cancer in Young Adults and Nonsmokers: Study of Cancer Susceptibility by Genome‐Wide High‐Density SNP Microarray Mapping,” Acta Oto‐Laryngologica 131, no. 10 (2011): 1091–1098, 10.3109/00016489.2011.590151. 21631177

[3] S. Tengku , I. Lohi , A. Connelly , et al., “Late‐Onset Swallowing Outcomes Post‐Treatment for Head and Neck Cancer in a UK‐Based Population,” Journal of Laryngology and Otology 137, no. 3 (2023): 293–300, 10.1017/S0022215122000834. 35317872 PMC9975761

[4] D. Adelstein , M. L. Gillison , D. G. Pfister , et al., “NCCN Guidelines Insights: Head and Neck Cancers, Version 2.2017,” Journal of the National Comprehensive Cancer Network 15, no. 6 (2017): 761–770, 10.6004/jnccn.2017.0101. 28596256

[5] N. Sathish , X. Wang , and Y. Yuan , “Human Papillomavirus (HPV)‐Associated Oral Cancers and Treatment Strategies,” Journal of Dental Research 93, no. 7_suppl (2014): 29S–36S, 10.1177/0022034514527969. 24663683 PMC4107541

[6] S. Marur , G. D'Souza , W. H. Westra , and A. A. Forastiere , “HPV‐Associated Head and Neck Cancer: A Virus‐Related Cancer Epidemic,” Lancet Oncology 11, no. 8 (2010): 781–789, 10.1016/S1470-2045(10)70017-6. 20451455 PMC5242182

[7] F. Ngabo , S. Franceschi , I. Baussano , et al., “Human Papillomavirus Infection in Rwanda at the Moment of Implementation of a National HPV Vaccination Programme,” BMC Infectious Diseases 16, no. 1 (2016): 225, 10.1186/s12879-016-1539-6. 27221238 PMC4877733

[8] F. Sayinzoga , V. Tenet , D. A. M. Heideman , et al., “Human Papillomavirus Vaccine Effect Against Human Papillomavirus Infection in Rwanda: Evidence from Repeated Cross‐Sectional Cervical‐Cell‐Based Surveys,” Lancet Global Health 11, no. 7 (2023): e1096–e1104, 10.1016/S2214-109X(23)00193-6. 37207683 PMC10282073

[9] S. Okerosi , L. W. Mokoh , F. Rubagumya , et al., “Human Papillomavirus–Associated Head and Neck Malignancies in Sub‐Saharan Africa: A Systematic Review,” JCO Global Oncology 9 (2023): e2200259, 10.1200/GO.22.00259. 36730877 PMC10166441

[10] A. Atkinson , C. Studwell , S. Bejarano , et al., “Rural Distribution of Human Papilloma Virus in Low‐ and Middle‐Income Countries,” Experimental and Molecular Pathology 104, no. 2 (2018): 146–150, 10.1016/j.yexmp.2018.03.001. 29551573

[11] S. A. Turner , S. J. Deharvengt , K. D. Lyons , et al., “Implementation of Multicolor Melt Curve Analysis for High‐Risk Human Papilloma Virus Detection in Low‐ and Middle‐Income Countries: A Pilot Study for Expanded Cervical Cancer Screening in Honduras,” Journal of Global Oncology 4 (2017): JGO.17.00035, 10.1200/JGO.17.00035. 30241169 PMC6180764

[12] F. Kabagenyi , J. Otiti , J. Namwagala , A. Kamulegeya , and S. Kalungi , “A Descriptive Study of Human Papilloma Virus in Upper Aero‐Digestive Squamous Cell Carcinoma at Uganda Cancer Institute Assessed by P16 Immunohistochemistry,” Cancers Head Neck 5 (2020): 10, 10.1186/s41199-020-00057-3. 32864169 PMC7450959

[13] C. Ndiaye , L. Alemany , Y. Diop , et al., “The Role of Human Papillomavirus in Head and Neck Cancer in Senegal,” Infectious Agents and Cancer 8, no. 1 (2013): 14, 10.1186/1750-9378-8-14. 23594504 PMC3637397

[14] J. Nabukenya , T. A. Hadlock , and W. Arubaku , “Head and Neck Squamous Cell Carcinoma in Western Uganda: Disease of Uncertainty and Poor Prognosis,” OTO Open 2, no. 1 (2018): 2473974X18761868, 10.1177/2473974X18761868. PMC6239026 30480207

[15] G. Shrestha , C. P. Chang , C. B. Pun , et al., “Differences in Risk Factors for Head and Neck Cancer Among Men and Women in Nepal: A Case‐Control Study,” Cancer Epidemiology 82 (2023): 102319, 10.1016/j.canep.2022.102319. 36566578 PMC9852028

[16] J. Aswani , O. Anzala , and N. Mwang'ombe , “High Risk Human Papillomavirus in Head and Neck Squamous Cell Carcinoma Patients at Kenyatta National Hospital, Kenya,” African Journal of Health Sciences 32, no. 2 (2019): Art. no. 2, 10.4314/ajhs.v32i2.

[17] M. Mourad , T. Jetmore , A. A. Jategaonkar , S. Moubayed , E. Moshier , and M. L. Urken , “Epidemiological Trends of Head and Neck Cancer in the United States: A SEER Population Study,” Journal of Oral and Maxillofacial Surgery 75, no. 12 (2017): 2562–2572, 10.1016/j.joms.2017.05.008. 28618252 PMC6053274

[18] B. A. Mahal , P. J. Catalano , R. I. Haddad , et al., “Incidence and Demographic Burden of HPV‐Associated Oropharyngeal Head and Neck Cancers in the United States,” Cancer Epidemiology, Biomarkers & Prevention 28, no. 10 (2019): 1660–1667, 10.1158/1055-9965.EPI-19-0038. 31358520

[19] C. Faggons , C. Mabedi , C. Shores , and S. Gopal , “Review: Head and Neck Squamous Cell Carcinoma in Sub‐Saharan Africa,” Malawi Medical Journal 27, no. 3 (2015): 79–87. 26715951 10.4314/mmj.v27i3.2 PMC4688867

[20] C. Ndiaye , M. Mena , L. Alemany , et al., “HPV DNA, E6/E7 mRNA, and p16INK4a Detection in Head and Neck Cancers: A Systematic Review and Meta‐Analysis,” Lancet Oncology 15, no. 12 (2014): 1319–1331, 10.1016/S1470-2045(14)70471-1. 25439690

[21] X. Castellsagué , L. Alemany , M. Quer , et al., “HPV Involvement in Head and Neck Cancers: Comprehensive Assessment of Biomarkers in 3680 Patients,” Journal of the National Cancer Institute 108, no. 6 (2016): djv403, 10.1093/jnci/djv403. 26823521

[22] T. R. Sekee , F. J. Burt , D. Goedhals , J. Goedhals , Y. Munsamy , and R. Y. Seedat , “Human Papillomavirus in Head and Neck Squamous Cell Carcinomas in a South African Cohort,” Papillomavirus Research 6 (2018): 58–62, 10.1016/j.pvr.2018.10.006. 30391364 PMC6232649

[23] M. Brunner , O. Koperek , F. Wrba , et al., “HPV Infection and p16 Expression in Carcinomas of the Minor Salivary Glands,” European Archives of Oto‐Rhino‐Laryngology 269, no. 10 (2012): 2265–2269, 10.1007/s00405-011-1894-2. 22207527

[24] A. Takla , M. Wiese‐Posselt , T. Harder , et al., “Background Paper for the Recommendation of HPV Vaccination for Boys in Germany,” Bundesgesundheitsblatt ‐ Gesundheitsforschung ‐ Gesundheitsschutz 61, no. 9 (2018): 1170–1186, 10.1007/s00103-018-2791-2. 30167729

[25] K. Schneider , C. Grønhøj , C. H. Hahn , and C. von Buchwald , “Therapeutic Human Papillomavirus Vaccines in Head and Neck Cancer: A Systematic Review of Current Clinical Trials,” Vaccine 36, no. 45 (2018): 6594–6605, 10.1016/j.vaccine.2018.09.027. 30268734

[26] G. D'Souza and A. Dempsey , “The Role of HPV in Head and Neck Cancer and Review of the HPV Vaccine,” Preventive Medicine 53 (2011): S5–S11, 10.1016/j.ypmed.2011.08.001. 21962471 PMC3287051

